# Evaluation of NPH Insulin Dosing Interval for Critically Ill Hyperglycemic Trauma Patients During Continuous Enteral Nutrition: A Pilot Study

**DOI:** 10.3390/nu17172880

**Published:** 2025-09-05

**Authors:** Delaney S. Adams, Brandon D. Conaway, Julie E. Farrar, Saskya Byerly, Dina M. Filiberto, Roland N. Dickerson

**Affiliations:** 1 Department of Clinical Pharmacy and Translational Science, College of Pharmacy, University of Tennessee Health Science Center, Memphis, TN 38163, USA; dadams1@regionalonehealth.org (D.S.A.); bdconaway@gmail.com (B.D.C.); jfarrar7@uthsc.edu (J.E.F.); 2 Department of Pharmacy, Regional One Health, Memphis, TN 38103, USA; 3 Department of Surgery, College of Medicine, University of Tennessee Health Science Center, Memphis, TN 38163, USA; sbyerly1@uthsc.edu (S.B.); dfiliber@uthsc.edu (D.M.F.)

**Keywords:** adult, critical care, diabetes, nutrition, trauma, enteral nutrition, insulin

## Abstract

Objective: The aim of this study was to retrospectively evaluate the results of administering subcutaneous neutral protamine Hagedorn (NPH) insulin every 8 h (NPH-8) versus every 12 h (NPH-12) in critically ill, hyperglycemic trauma patients who required continuous enteral nutrition (EN). Methods: Both groups of patients were given concurrent sliding scale regular human insulin (SSI) with NPH therapy. The evaluation of glycemic control continued for 7 days. Results: A total of 15 patients were given NPH every 8 h (NPH-8), and 19 were given NPH every 12 h (NPH-12). Carbohydrate intake was similar between groups (115 ± 35 vs. 108 ± 37 g/d; *p* = 0.584). There was no significant difference in average blood glucose (BG) concentration (168 ± 18 vs. 166 ± 17 mg/dL; *p* = 0.803) or time within a BG target range of 70 to 149 mg/dL (7.5 ± 4.7 vs. 8.1 ± 5.0 h/d; *p* = 0.678) or 70 to 179 mg/dL (14.5 ± 5.0 vs. 16 ± 5.6 h/d; *p* = 0.419) or the incidence of Level 1 hypoglycemia (2 patients in each group; *p* = 1.00) or Level 2 hypoglycemia (1 patient vs. 0 patients, *p* = 0.441) between the NPH-8 and NPH-12 groups, respectively. However, the NPH-8 group required twice as much total (NPH + SSI) insulin (115 ± 52 vs. 58 ± 33 units/d; *p* = 0.004). Conclusions: These preliminary data suggest no significant difference between the administration of NPH-8 and NPH-12 based on glycemic control metrics in critically ill hyperglycemic trauma patients given EN. However, these results may be confounded by a selection bias as to who received NPH-8 vs. NPH-12. Further research is required.

## 1. Introduction

Critically ill trauma patients are susceptible to hyperglycemia due to insulin resistance and accelerated gluconeogenesis from increased cytokine and counter-regulatory hormone production including endogenous and exogenous catecholamines and corticosteroid administration [1]. This altered glycemic regulation can be further magnified by the concurrent provision of nutrition therapy and the presence of diabetes, particularly if diabetes was uncontrolled prior to admission to the trauma intensive care unit (TICU) [2]. Because hyperglycemia has been associated with poorer clinical outcomes in critically ill trauma patients [3,4,5,6,7], significant efforts have been implemented to improve appropriate glycemic control for this population [8,9,10]. Although a continuous intravenous (IV) regular human insulin (RHI) infusion provides the most precise glycemic control [1,9], its implementation requires substantial nursing labor as blood glucose concentration (BG) monitoring and continuous IV RHI dosage titration occur on an hourly basis. During nursing staffing shortages or in environments with limited resources, it may be desirable to use intermediate-acting insulin therapy (e.g., neutral protamine Hagedorn (NPH) insulin) with supplemental RHI with BG monitoring every 3 to 4 h as opposed to a continuous IV RHI infusion with hourly BG monitoring when feasible. However, because over half of critically ill trauma patients experience augmented renal clearance [11], insulin clearance may be increased and have a shorter duration of action in some patients. Thus, the conventional 12 h dosing of NPH insulin may be insufficient to maintain glycemic control and potentially result in more glycemic variability [12,13].

The aim of this study was to ascertain whether there is an advantage of administering subcutaneous NPH insulin every 8 h versus every 12 h in critically ill, hyperglycemic trauma patients who required continuous enteral nutrition (EN).

## 2. Materials and Methods

**Figure 1:**
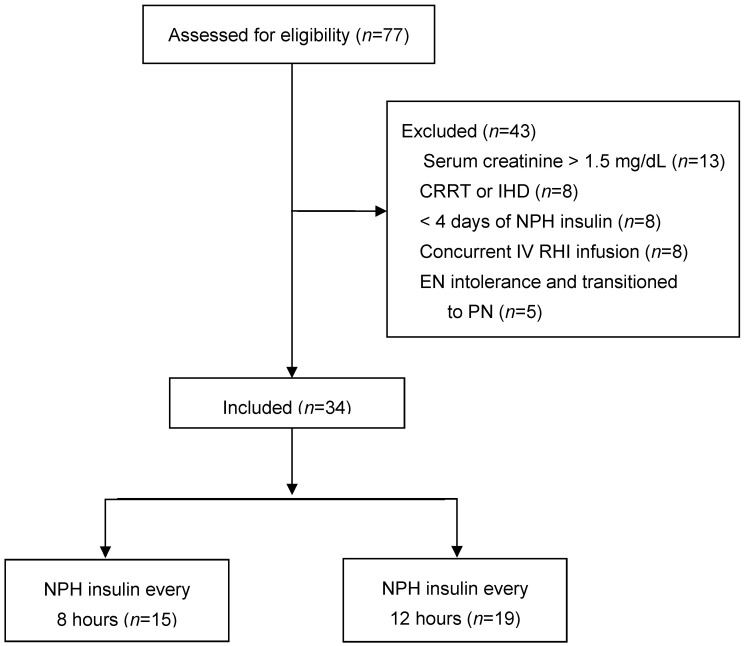
Patient selection and classification by NPH dosing interval. CRRT, continuous renal replacement therapy; EN, enteral nutrition; IHD, intermittent hemodialysis; IV, intravenous; *n*, number of patients; NPH, neutral protamine Hagedorn insulin; PN, parenteral nutrition; RHI, regular human insulin.

Adult patients, greater than 17 years of age, admitted to the TICU from April 2020 to August 2023, who received continuous EN and followed by the Nutrition Support Service (NSS) and who exhibited a BG greater than 179 mg/dL (9.9 mmol/L) necessitating intensive insulin therapy were retrospectively identified for this study. Intensive insulin therapy was defined by the implementation of subcutaneous NPH insulin with intravenous sliding scale regular human insulin (SSI) or a continuous IV RHI infusion with transition to NPH with SSI. Patients were excluded if they received less than 4 days of NPH, an IV RHI infusion during NPH, or parenteral nutrition. Other exclusion criteria were those with kidney failure, EN intolerance, or other conditions, as given in Figure 1.

The evaluation of glycemic control began the day before starting NPH therapy and continued for up to 7 days while receiving NPH insulin during continuous EN. The primary target BG range was 70 to 150 mg/dL (3.9 to 8.3 mmol/L) [2,8,9,10,14], which is similar to the recently published American Association for the Surgery of Trauma/American College of Surgeons Committee’s recommendation of 80 to 150 mg/dL (4.4 to 8.3 mmol/L) for critically ill patients with traumatic injuries [1]. A secondary target BG range of 70 to 179 mg/dL (3.8 to 9.9 mmol/L) was evaluated with the upper limit BG concentration based on the American Diabetes Association consensus recommendations [15]. Home pharmacotherapy for the management of diabetes was suspended upon TICU admission.

Patients were prescribed NPH insulin if they exhibited three BGs ≥ 150 mg/dL (8.3 mmol/L) within 24 h while receiving EN based on the judgment of NSS personnel and the TICU attending physician. Patients were given subcutaneous NPH insulin every 8 or 12 h at the discretion of the NSS in consultation with the primary trauma service and were evaluated up to the first 7 days of NPH therapy. Patients were initially given a total NPH dose of ~40% to 50% of the total RHI dose given on the previous day and titrated upwards thereafter [10,14]. NPH dosage also increased proportionately with the increase in carbohydrate intake along with the consideration of the amount of required insulin via SSI [10,14]. SSI doses were given according to the algorithm given in Table 1 [8]. BG determinations when using SSI were obtained every 3 or 4 h. A large-volume IV solution containing 5% dextrose was infused at the same rate as the continuous EN in the event that EN was abruptly discontinued. The evening dose of NPH insulin was withheld prior to any scheduled operative procedures for the following day and was restarted postoperatively following the resumption of continuous EN.

Point-of-care BG measurements were performed using StatStrip^®^ (Nova Biomedical, Waltham, MA, USA). Blood for the BG measurement was obtained via a capillary fingerstick or by central venous access at the discretion of the TICU nurse depending on the availability of a catheter port with respect to the timing of BG determination and the use of the catheter port for intravenous infusions and medications. Level 1 and Level 2 hypoglycemia was defined as BG < 70 mg/dL (3.9 mmol/L) but ≥54 mg/dL (3 mmol/L) and <54 mg/dL (3 mmol/L), respectively [16]. Patients who experienced hypoglycemia were given either 12.5 g or 25 g of intravenous dextrose irrespective of a lack of symptoms.

A caloric goal of 30 to 32 kcal/kg/d [17] and a protein goal of 2 to 2.5 g/kg/d [18] were assigned to patients without obesity (body mass index (BMI) < 30 kg/m^2^). Patients with obesity (BMI ≥ 30 kg/m^2^) were assigned a caloric goal of approximately 25 kcal/kg ideal body weight (IBW)/d and a protein goal of 2 to 2.5 g/kg IBW/d [19]. Patients not receiving propofol or without obesity were given a 1.2 kcal/mL, 60 g of protein per liter “diabetic” enteral formula containing 83 g of carbohydrate per 1000 kcals along with sugar-free liquid protein supplement boluses. Patients with obesity or those given a significant amount of calories via propofol [20] were given a 1 kcal/mL, 92 g of protein per L enteral formula containing 76 g of carbohydrate per 1000 kcals with or without the addition of sugar-free liquid protein supplements. Intragastric EN was preferentially given and initiated at a rate of 25 to 30 mL/h when the patient was hemodynamically stable. EN was advanced daily depending on enteral feeding tolerance, severity of glycemic excursions, or significant decreases in serum potassium and phosphorus concentrations.

The patients’ electronic health records were retrospectively reviewed for the collection of laboratory and clinical data. Serum chemistries were obtained daily. Plasma Hgb A_1c_ was obtained near admission for patients with a history of diabetes or those who were experiencing hyperglycemia. Hgb A_1c_ was determined by the hospital laboratory using a National Glycohemoglobin Standardization Program-certified immunoassay.

The mean daily BG level was calculated by taking the average of the number of BG determinations performed each day irrespective of any varying amounts of determinations for that day. The number of hours that the BG level was above, below, and within the target BG range was calculated by assuming that the BG level remained constant during that time interval until the next BG determination. None of the daily BG and hours in BG range data were interpolated. Because many medical and mixed medical/surgical intensive care units target a higher BG threshold than 149 mg/dL [21], the number of hours that the BG level was above, below, and within 70 to 179 mg/dL (3.9 mmol/L to 9.9 mmol/L) was also evaluated. The number of episodes and severity of hypoglycemia were also noted. The coefficient of variation in daily BG measurements was used to assess glycemic variability. Daily carbohydrate intake was calculated from the electronic nursing records of EN volume intake and supplemental dextrose-containing IV fluids. The amounts of NPH and SSI given daily were also obtained from the hospital electronic records.

Data were analyzed using SigmaPlot for Windows version 11.2 (Systat Software, Point Richmond, CA, USA). Interval data were reported as the mean ± SD. Differences between groups were evaluated by Student’s *t*-test or the Wilcoxon rank sum test. Chi-Square analysis or Fischer’s Exact test were used to compare nominal data. Multiple serial measurements for the NPH dosing groups were analyzed via a two-way analysis of variance with post hoc pairwise comparisons using the Student–Newman–Keuls test. Statistical significance was defined as a *p* value of < 0.05. This study was approved by the University IRB and the Hospital Office for Medical Research. The requirement for informed consent was waived as all measurements were performed as part of the routine clinical care of the patients, the data was collected retrospectively, and confidentiality procedures for the patients were maintained.

## 3. Results

Seventy-five critically ill, hyperglycemic patients who received NPH and were referred to the NSS for continuous EN were initially evaluated. Most of the forty-three patients that were excluded were excluded due to kidney failure or the fact that they received <4 days of NPH or received concurrent continuous IV RHI infusion at the same time as NPH (Figure 1). Of the 34 evaluable patients, 15 were given NPH every 8 h (NPH-8), and 19 were given NPH every 12 h (NPH-12). SSI was given to both groups. Most of the patients were male (71%), admitted to the TICU because of a motor vehicle collision (79%), and ventilator-dependent (94%). Many patients were middle-aged or older (58 ± 13 years), had an overweight or obese BMI (36.3 ± 9.1 kg/m^2^), had diabetes (85%), and received insulin therapy at home prior to admission (32%). There were no statistically significant differences between groups in age, race, admission diagnosis, ventilator dependence, vasopressor or corticosteroid therapy, or inflammatory markers such as C-reactive protein concentration, prealbumin concentration, white blood cell count, or maximum body temperature (Table 2). However, the NPH-8 group exhibited a greater body mass index and tended to have more patients with traumatic brain injury than the NPH-12 group. Fifteen patients (44%) had a baseline Hgb A_1c_ ≥ 8.0% (44% of NPH-8 patients and 42% of NPH-12 patients). Other demographic data are provided in Table 2.

The maximum BG before the initiation of insulin therapy approached 300 mg/dL (16.7 mmol/L) for the NPH-8 group and was significantly greater than that for the NPH-12 group at ~250 mg/dL (13.9 mmol/L) (*p* = 0.046; Table 3). Prior to NPH therapy, three fourths of patients in the NPH-8 group were given a continuous intravenous RHI infusion compared to one third of patients in the NPH-12 group (*p* = 0.038). The NPH-8 group received over twice as much RHI as the NPH-12 group prior to NPH therapy (*p* = 0.001; Table 3).

**Figure 2:**
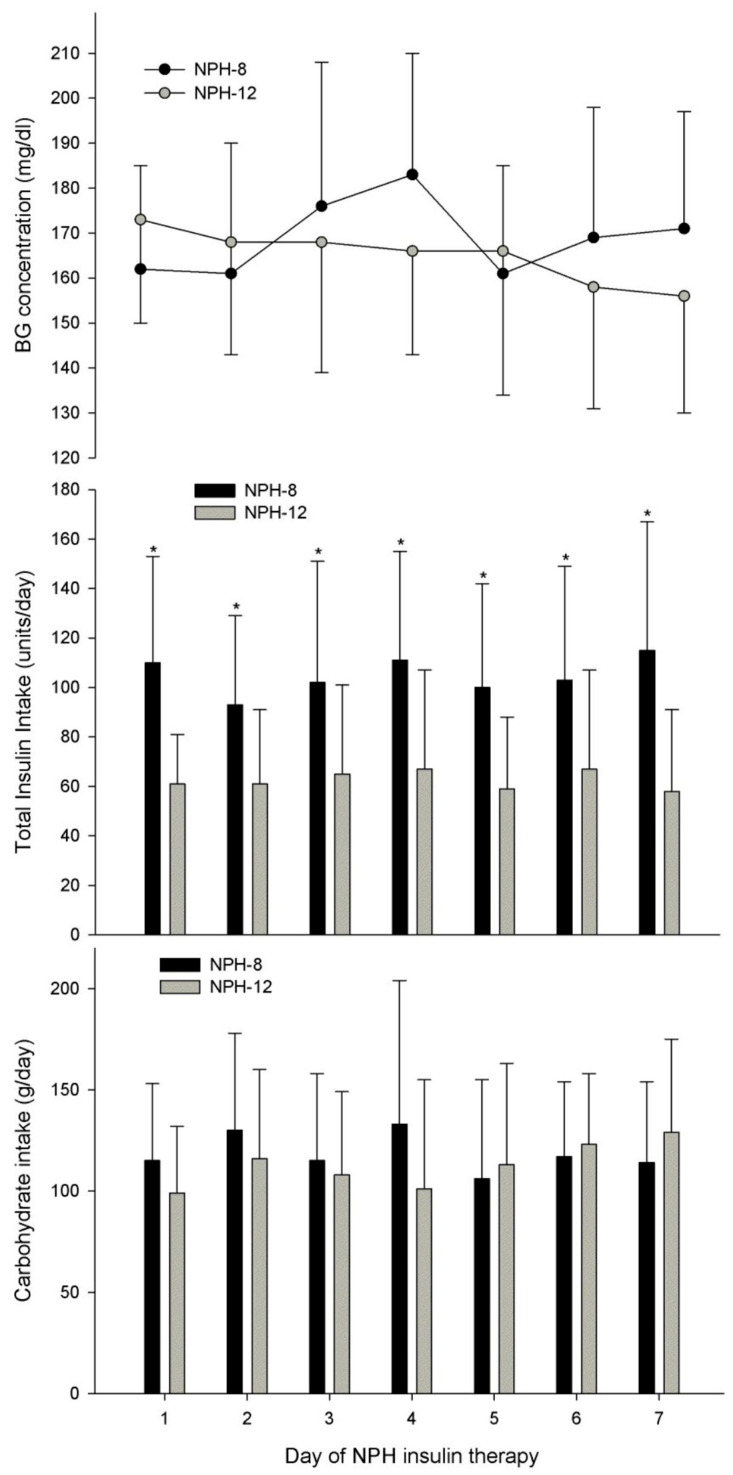
Blood glucose response to total insulin therapy (NPH and SSI) and carbohydrate intake in patients who received NPH insulin every 8 h versus every 12 h. No significant difference was noted for daily blood glucose concentrations (*p* = 0.271) and carbohydrate intakes (*p* = 0.772) between NPH dosing interval groups. Total insulin intake was significantly greater for those who received NPH insulin every 8 h (NPH-8) versus every 12 h (NPH-12, *p* < 0.001) with significant differences (*p* < 0.05) in total insulin intakes between groups for each day during observation period. Blood glucose concentration (mmol/L) = blood glucose concentration (mg/dL) ÷ 18. NPH, neutral protamine Hagedorn insulin; SSI, sliding scale regular human insulin. * *p* < 0.05 between groups for the day.

**Figure 3:**
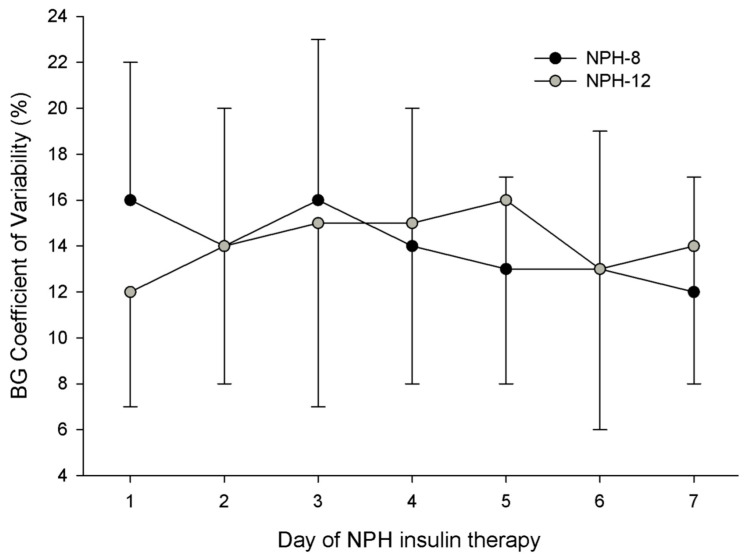
Variability in blood glucose control, as defined by the coefficient of variation, for the NPH-8 and NPH-12 groups. There was no significant difference between groups for the observation period (*p* = 0.908). NPH, neutral protamine Hagedorn insulin; NPH-8, the group given NPH insulin every 8 h; NPH-12, the group given NPH insulin every 12 h.

There was no statistically significant difference in average daily BG, time within or above the target ranges, or the incidence of Level 1 or 2 hypoglycemia between groups during the 7-day observation period (Table 3, Figure 2). BG variability was similar between the NPH-8 and NPH-12 groups (Table 3, Figure 3). Despite similar daily carbohydrate intakes between the NPH-8 and NPH-12 groups (115 ± 35 vs. 108 ± 37 g/d, respectively; *p* = 0.584), the NPH-8 group required a significantly greater total (NPH and SSI) daily insulin intake than the NPH-12 group (115 ± 35 vs. 58 ± 15 units/d, respectively; *p* = 0.004; Table 3, Figure 2). This difference in total daily insulin intake between groups was further defined by the receipt of twice as much daily NPH insulin in the NPH-8 group (62 ± 30 vs. 26 ± 15 units/d, respectively; *p* = 0.006) as SSI intake was similar between groups (42 ± 18 vs. 35 ± 15 units/d, respectively; *p* = 0.206; Table 3). The NPH-8 group also required, on average, a five day longer duration of NPH therapy than the NPH-12 group (*p* = 0.006; Table 3).

Survival and infection rates were similar between groups, whereas hospital and TICU length of stay tended to be greater for the NPH-8 group (Table 2). However, the assessment of these clinical outcomes is difficult due to insufficient statistical power.

## 4. Discussion

These data indicated that NPH-8 was not more effective than NPH-12 in improving glycemic control as evidenced by an equivalent average BG, number of hours within the 70 to 149 mg/dL (3.9 to 8.3 mmol/L) and 70 to 179 mg/dL (3.9 to 9.9 mmol/L) target BG ranges, and variability during the 7-day observation period. Safety levels between both treatment arms were also similar as 11% and 13% of the NPH-8 and NPH-12 groups experienced an episode of Level 1 hypoglycemia, and one patient versus no patients experienced an episode of Level 2 hypoglycemia, respectively.

The efficacy and safety of a therapeutic regimen for glycemic control entails the evaluation of hyperglycemia, hypoglycemia, and glycemic variability [12]. It is important to reduce hyperglycemia in critically ill trauma patients as it is associated with increased morbidity and mortality [3,6]. The American Diabetic Association (ADA) Professional Practice Committee [15] suggests a target BG range of 140 to 180 mg/dL (7.8 to 10 mmol/L) for most critically ill patients based on the NICE-SUGAR trial, in which many participants were medical intensive care unit patients [21]. However, the ADA Professional Practice Committee acknowledges that more stringent individualized glycemic goals may be appropriate for selected critically ill populations if they can be safely achieved without significant hypoglycemia [15]. Studies in critically ill trauma patients have indicated that a target BG of less than 140 to 150 mg/dL (8.1 to 8.3 mmol/L) improved morbidity and mortality [5,7,22]. Based on these studies, a 2023 consensus statement from the American Association for the Surgery of Trauma/American College of Surgeons [1] recommends a target BG of 80 to 150 mg/dL (4.4 to 8.3 mmol/L). A potential argument for less strict glycemic control pertains to medical and surgical patients with chronically uncontrolled diabetes (defined by a Hgb A_1c_ ≥ 8.0%) as worsened outcomes were not associated with hyperglycemia [23,24,25,26,27]. It has been inferred that the lack of association between hyperglycemia and mortality in chronically uncontrolled diabetic patients may be due to their adaptation to chronic hyperglycemia, glycemic excursions, and variability [27]. Because of this controversy and because 44% of patients had a Hgb A_1c_ ≥ 8.0%, we assessed glycemic control using two target BG ranges: 70 to 149 mg/dL (3.9 to 8.3 mmol/L) and 70 to 179 mg/dL (3.9 to 9.9 mmol/L). Time in the tighter BG target range averaged about 8 h per day for both groups and 15 to 16 h per day for a BG target < 180 mg/dL (10 mmol/L) for the NPH-8 and NPH-12 groups, respectively. Safety was reasonable since only 13% and 11% of patients experienced an episode of Level 1 hypoglycemia. One patient from the NPH-8 group experienced Level 2 hypoglycemia. No adverse consequences from the hypoglycemic events were reported. Despite the infrequency of hypoglycemia and irrespective of a lack of mortality associations with hypoglycemia in some studies [25], preventing hypoglycemia for patient safety is of utmost importance with insulin therapy and glycemic control procedures.

Long-acting insulin analogs such as insulin glargine or detemir may reduce glycemic variability due to their flattened peak and nadir pharmacokinetic profiles. However, we preferred intermediate-acting NPH insulin as EN can be interrupted, sometimes for an extended time, due to surgical procedures and feeding intolerance in critically ill patients with traumatic injuries [10,28,29]. Thus, the intent for using intermediate-acting NPH with SSI was to mitigate potential hypoglycemic episodes that might occur from the use of prolonged-acting insulin during the absence of EN as described by others [30,31]. The administration of NPH insulin every 8 h as opposed to every 12 h was anticipated to dampen peak and nadir insulin concentrations, thereby potentially reducing glycemic variability and the occurrence of hypoglycemia.

Unfortunately, no difference in glycemic variability, time in the target BG range, or incidence of hypoglycemia was observed between the two dosing intervals. However, patients who received dosing every 8 h had a greater BG level prior to insulin therapy, and nearly three fourths of patients required a continuous IV RHI infusion compared to one third of the NPH-12 group prior to NPH therapy (Table 3). Also, the NPH-8 group received over twice as much RHI prior to the initiation of NPH (Table 3). NSS personnel, upon reviewing the patients’ glycemic control challenges for those who required larger amounts of RHI prior to NPH, likely biased the dosing interval selection towards a dosing interval of every 8 h based on the empiric assumption of wide BG variability and low BG nadirs with a dosing strategy of every 12 h in this subgroup of patients. This is evidenced by the receipt of an average of 63 units per day of NPH (about 20 units three times daily) for the NPH-8 group compared to 26 units per day for the NPH-12 group (Table 3). Thus, the NPH-8 group would have received an average of 32 units twice daily if the dosing strategy of every 12 h was used compared to an average of 13 units twice daily received by the NPH-12 group.

Another paramount strategy to address glycemic control for critically ill hyperglycemic patients is to provide a more restricted carbohydrate intake by the use of enteral formulas lower in carbohydrate content than standard formulas as glycemic control is difficult to achieve with higher-carbohydrate-containing enteral formulas [32,33]. In addition, the use of non-dextrose-containing IV fluids whenever possible was also undertaken. As a result of these efforts, we were able to restrict the mean total carbohydrate intakes for both groups to about 110 to 115 g per day (Table 3).

No statistically significant differences in any of the clinical outcomes including survival and the development of infections occurred between groups. TICU and hospital lengths of stay for the NPH-8 group were longer by 8 and 9 days (Table 2), respectively, but these trends were statistically insignificant due to considerable variability in a limited number of patients. It may be hypothesized that this trend in the greater length of ICU and hospital stays for the NPH-8 group may be tangential to requiring twice as much insulin to achieve glycemic control despite lacking differences in Injury Severity Scores, inflammatory markers, presence of diabetes or obesity, carbohydrate intake, or pharmacotherapy known to worsen hyperglycemia (Table 3). However, further confirmatory research of this observation in a large population is warranted. The strengths of this study include that patients were managed by a multidisciplinary nutrition support team that was intimately engaged in nutrition therapy and glycemic control with the use of standardized insulin treatment algorithms in consultation with the trauma intensive care unit service. This team care approach facilitated continuity in patient care. The limitations of this study include its retrospective study design. The limited number of patients diminishes statistical power for identifying nuanced differences between cohorts. Also, potential prescriber preferences for the selection of who received NPH dosing every 8 h versus every 12 h resulted in a population selection bias, with those requiring the most insulin being placed into the group receiving the 8 h dosing strategy. The recent 2023 American Association for the Surgery of Trauma/American College of Surgeons recommendations [1] indicate a minimum threshold BG of 80 mg/dL (4.4 mmol/L); however, we used 70 mg/dL (3.9 mmol/L) since hypoglycemia is defined as a level <70 mg/dL (3.9 mmol/L) per the American Diabetes Association [15]. The 7-day observation period potentially limits the ability to fully evaluate the long-term clinical impact of the dosing strategies. The single-center nature of this study limits its generalizability to other critically ill patient populations. Finally, it is imperative to note that there was selection bias in those who received NPH insulin every 8 h versus every 12 h as the NPH-8 population, despite receiving equivalent amounts of carbohydrate, required about twice as much insulin to achieve equivalent glycemic control. This pilot study indicates the requirement for a future prospective randomized controlled trial comparing the two dosing strategies to ensure equivalent populations between groups.

## 5. Conclusions

These hypothesis-generating preliminary data suggest no significant difference between the administration of NPH insulin every 8 h compared to every 12 h based on glycemic control metrics in critically ill hyperglycemic trauma patients given EN. However, the NPH-8 group had significantly higher insulin requirements at baseline and during NPH therapy than the NPH-12 group despite similar carbohydrate intakes. These findings indicate that the NPH-8 group was a more challenging glycemic control population reflective of differences in baseline population characteristics and not necessarily because of the dosing interval itself. Further research via a randomized controlled trial is required to ascertain whether similar glycemic control metrics occur when both dosing strategies are compared within a similar challenging hyperglycemic population.

## Figures and Tables

**Table 1:** Sliding scale RHI algorithms.

BG, mg/dL (mmol/L)	Lower-Intensity SSI Intervention	Higher-Intensity SSI Intervention
<40 (2.2)	Give 25 g D50W IV; call primary service	Give 25 g D50W IV; call primary service
40–70 (2.2–3.8)	Give 12.5 g D50W IV; call primary service	Give 12.5 g D50W IV; call primary service
71–125 (3.9–6.9)	0 units RHI	0 units RHI
126–150 (7.0–8.3)	2 units RHI	3 units RHI
151–175 (8.4–9.7)	4 units RHI	6 units RHI
176–200 (9.8–11.1)	6 units RHI	9 units RHI
201–225 (11.2–12.5)	8 units RHI	12 units RHI
226–250 (12.6–13.8)	10 units RHI	15 units RHI
251–275 (13.9–15.2)	12 units RHI	18 units RHI
276–300 (15.3–16.6)	14 units RHI	21 units RHI
>300 (16.6)	16 units RHI; call primary service	24 units RHI; call primary service

BG, blood glucose concentration; D50W, 50% dextrose in water; IV, intravenous; RHI, regular human insulin; SSI, sliding scale regular human insulin.

**Table 2:** Patient characteristics and clinical outcomes.

Variable *	NPH-8	NPH-12	*p*
*n*	15	19	-
Age, y	54 ± 12	61 ± 14	0.127
Sex, male/female	11/4	13/6	1.000
Race White Black Hispanic	8 7 0	14 4 1	0.220
Admission diagnosis MVC, *n* GSW, *n* Fall/Assault, *n* Other, *n*	13 0 1 1	14 1 4 0	0.332
Injury severity score	22.7 ± 10.3	22.7 ± 9.3	0.996
Ventilator-dependent, *n* (%)	14 (93%)	18 (95%)	1.000
TBI, *n* (%)	11 (67%)	7 (37%)	0.077
Vasopressor therapy, *n* (%)	1 (7%)	1 (5%)	1.000
Corticosteroid therapy, *n* (%)	1 (7%)	3 (16%)	0.613
Weight, kg	119 ± 24	100 ± 33	0.065
BMI, kg/ht^2^	38.1 ± 5.8	34.8 ± 11.0	0.048
Prealbumin, mg/dL	9.5 ± 4.2	7.7 ± 3.4	0.189
C-reactive protein, mg/dL	24.1 ± 6.6	24.5 ± 9.1	0.873
WBC, cells/µm^3^	12.2 ± 3.9	12.9 ± 6.5	0.703
Tmax, °C	37.7 ± 0.5	37.7 ± 0.5	0.854
Serum creatinine, mg/dL	1.1 ± 0.3	0.9 ± 0.3	0.092
Home insulin therapy, *n* (%)	5 (33%)	6 (32%)	0.616
Diabetes, *n* (%)	12 (80%)	17 (89%)	0.634
Hgb A_1c_, %	8.6 ± 2.3	7.6 ± 1.4	0.159
Hospital day of Hgb A_1c_, d	3 ± 3	5 ± 5	0.284
Survived, *n* (%)	14 (97%)	18 (95%)	1.000
Infection, *n* (%)	7 (47%)	10 (53%)	1.000
TICU LOS, d	30 ± 29	22 ± 15	0.340
Hospital LOS, d	42 ± 31	33 ± 17	0.445

* BMI, body mass index; GSW, gunshot wound; Hgb, hemoglobin; LOS, length of stay; MVC, motor vehicle collision; *n*, number of patients; TBI, traumatic brain injury; TICU, trauma intensive care unit; Tmax, maximum temperature; WBC, white blood cell count; y, years.

**Table 3:** Glycemic control metrics.

Variable *	NPH-8	NPH-12	*p*
*n*	15	19	-
Max BG prior to RHI, mg/dL mmol/L	293 ± 82 16.3 ± 4.6	248 ± 97 13.8 ± 5.4	0.046
IV RHI infusion prior to NPH, *n* (%)	11 (73%)	6 (32%)	0.038
NPH intake, units/d	62 ± 30	26 ± 15	0.026
NPH duration, d	13 ± 7	8 ± 5	0.006
SSI intake, units/d	42 ± 18	35 ± 15	0.206
Total insulin intake, units/d	115 ± 52	58 ± 33	0.004
CHO intake, g/d	115 ± 35	108 ± 37	0.584
RHI received prior to NPH, units/d	97 ± 35	44 ± 25	0.001
BG 70–149 mg/dL, h/d 3.9–8.3 mmol/L	7.5 ± 4.7	8.1 ± 5.0	0.678
BG > 149 mg/dL, h/d >8.3 mmol/L	16.5 ± 4.7	15.8 ± 5.0	0.703
BG 70–179 mg/dL, h/d 3.9–9.9 mmol/L	14.5 ± 5.0	16.0 ± 5.6	0.419
BG > 179 mg/dL, h/d >9.9 mmol/L	9.4 ± 5.0	8.0 ± 5.6	0.436
Level 1 hypoglycemia, *n* (%)	2 (13%)	2 (11%)	1.000
Level 2 hypoglycemia, *n* (%)	1 (5%)	0 (0%)	0.441
BG variability (CV), %	14.2 ± 3.4	13.9 ± 3.8	0.848

* BG, blood glucose concentration; CHO, carbohydrate; CV, coefficient of variation; d, day; h, hours; IV, intravenous; *n*, number of patients; NPH, neutral protamine Hagedorn insulin; Max, maximum; RHI, regular human insulin; RHI, regular human insulin; SSI, sliding scale RHI.

## Data Availability

The dataset used and analyzed in the current study is available from the corresponding author upon reasonable request.
